# Rapid progression of calcified nodules with increased lipid core burden in the right coronary artery

**DOI:** 10.1007/s12928-023-00911-4

**Published:** 2023-01-28

**Authors:** Takeshi Nishi, Yoshitaka Sasahira, Teruyoshi Kume, Satoshi Koto, Shiro Uemura

**Affiliations:** Department of Cardiology, Kawasaki Medical School, 577 Matsushima, Kurashiki, Okayama 701-0192 Japan

**Fig. 1:**
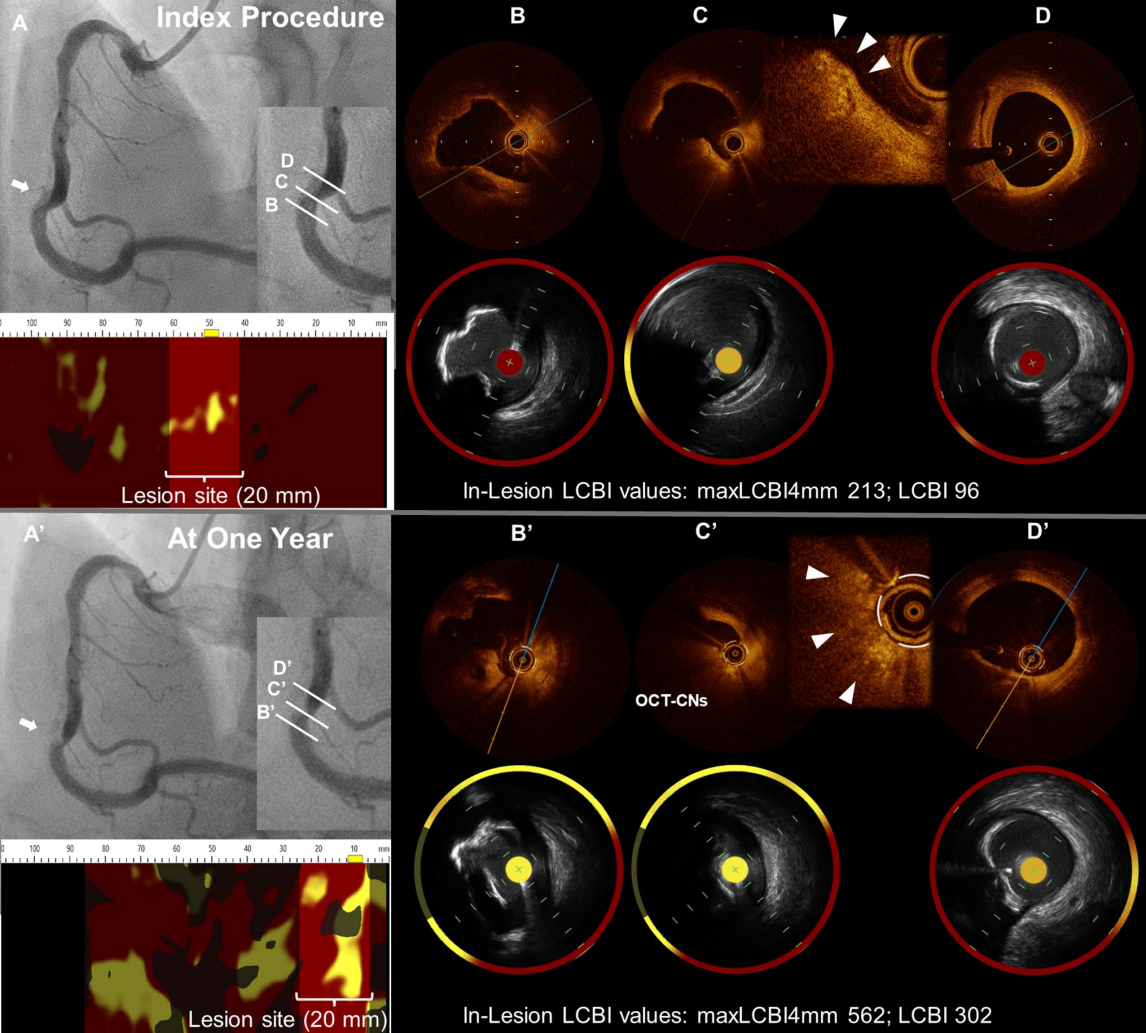
Serial coronary angiography (Panels **A** and **A**’) and intracoronary imaging (optical coherence tomography [OCT] and near-infrared spectroscopy [NIRS]-intravascular ultrasound) (Panels **B**,**D** and **B**’,**D**’) at the index procedure and 1 year.  The angiograms show a progression of a stenotic lesion in the mid right coronary artery in 1 year (white arrow, Panel **A** and **A**’). Panel **C** shows a protruding eccentric lesion with clustered nodules of calcification (arrowheads in magnified images) and adjacent sheet calcification (Panels **B**, **B**’, **D**, and **D**’), indicating nodular calcification (NC). Panel **C**’ shows a progression of protruding NC, or possibly calcified nodules (CNs), compromising the lumen area. We defined this lesion as OCT-defined CNs because OCT could not strictly differentiate NC and CNs, given the difficulty of proving the presence or absence of adherent thrombus and overlying tissues. The maximum LCBI value of any 4-mm segment (maxLCBI4mm) and LCBI value within the lesion increased from 213 to 562 and 96 to 302, respectively

A 51-year-old man with a history of hypertension and dyslipidemia presented with stable angina and underwent successful percutaneous coronary intervention with stent implantation to the left anterior descending. To evaluate angiographically moderate lesions in the right coronary artery (RCA) (Fig. 1A), we performed intracoronary optical coherence tomography (OCT) and near-infrared spectroscopy (NIRS)–intravascular ultrasound (IVUS), identifying eccentric nodular calcification (NC) protruding to the lumen in the mid-RCA and adjacent sheet calcification proximal and distal to the NC (Fig. 1B–D). One year later, he had recurrent angina with a stress myocardial scintigraphy showing ischemia in the inferior left ventricular myocardium. Coronary angiography revealed severe stenosis in the mid-RCA (Fig. 1A’); OCT and IVUS showed significant luminal narrowing at the site of the NC (or OCT-defined calcified nodules [CNs]) (Fig. 1B’–D’), where lipid core burden index (LCBI) on NIRS significantly increased compared with 1 year earlier. The lesion was treated with coronary orbital atherectomy followed by balloon dilatation, resulting in a successful reduction of luminal narrowing. We avoided stenting because of concerns about potential stent-related future adverse events.

The mechanisms of the initiation and development of NC/CNs were not fully understood. Based on detailed histological characterizations of CNs at autopsy, CNs are hypothesized to be formed from a breakdown of calcified necrotic cores and develop between flanking areas of stable fibrocalcific plaques in coronary arteries in which hinge motion, or excessive torsion is observed, namely in the proximal to midportions of tortuous RCA [1]. In line with the previous investigations, the (OCT-defined) CNs in this case were observed in between sheet calcification in the tortuous mid-RCA and included lipid core on NIRS at the CN site. The new important finding of the serial OCT and NIRS-IVUS imaging in this case was that the rapid progression of the OCT-defined CNs was accompanied by a significant increase in LCBI on NIRS at the CN site, suggesting that underlying necrotic cores of CNs, albeit not extensive in the early stage, could further develop with lipid accumulation and immune cell infiltration due to continuous mechanical stress, leading to luminal narrowing. This finding provides new insights into the mechanisms of CN progression, indicating the possible involvement of an increase in the lipid-rich necrotic cores. Intense lipid-lowering and anti-inflammatory therapy may be therapeutic options to stabilize CNs.
